# Representation learning of single-cell RNA-seq data

**DOI:** 10.1261/rna.080889.125

**Published:** 2026-04

**Authors:** Constantin Ahlmann-Eltze, Florian Barkmann, Jan Lause, Valentina Boeva, Dmitry Kobak

**Affiliations:** 1 Cancer Institute, University College London, London WC1E 6DD, United Kingdom; 2 Institute for Machine Learning, Department of Computer Science, ETH Zurich, 8092 Zurich, Switzerland; 3 Hertie Institute for AI in Brain Health, University of Tübingen, 72076 Tübingen, Germany; 4 ETH AI Center, ETH Zurich, 8092 Zurich, Switzerland; 5 Swiss Institute for Bioinformatics, 1015 Lausanne, Switzerland; 6 Institut Cochin, INSERM U1016, CNRS UMR 8104, Université Paris-Cité, 75015 Paris, France

**Keywords:** single-cell RNA sequencing, representation learning, embeddings

## Abstract

Single-cell RNA sequencing (scRNA-seq) has become a cornerstone experimental technique in tissue biology, with gene expression data for over 100 million cells available in public repositories. The high dimensionality, sparsity, and technical noise inherent to scRNA-seq data have motivated the development of a broad spectrum of representation learning approaches. These methods learn compressed, lower-dimensional representations of single-cell transcriptomes that are meant to preserve essential variation while reducing noise, and can be used for clustering, visualization, trajectory inference, and other downstream tasks. Furthermore, methods have emerged that aim to integrate data from multiple experiments by learning a common latent representation. In this review, we frame factor models, autoencoders, contrastive learning approaches, and transformer-based foundation models as distinct instances of the representation learning paradigm for scRNA-seq. We provide a coherent taxonomy of these methods that articulates their conceptual foundations, shared assumptions, and key distinctions. We also discuss benchmarking and identify major challenges and open questions that will shape the future of the field.

## INTRODUCTION

Since its inception in 2009 (Tang et al. 2009), single-cell RNA sequencing (scRNA-seq) has become a foundational experimental technique in cellular biology. As the experimental methods advanced, the scale of data collection has grown rapidly (Svensson et al. 2018, 2020b), with modern experiments sometimes profiling millions of cells in parallel. Databases collecting scRNA-seq data from individual publications, such as those of the Broad Institute (Tarhan et al. 2023) and the Chan Zuckerberg Initiative (CZI Cell Science Program et al. 2025), have amassed sequencing data from over 100 million cells.

The starting point for scRNA-seq analysis is typically a matrix of nonnegative integer counts that denote the number of RNA molecules of each gene detected in a given cell. Important properties of scRNA-seq data sets are that they are typically very high-dimensional, since genomes can contain several tens of thousands of genes, and very noisy and sparse, since the number of RNA molecules per gene and cell fluctuates, and not all RNA molecules in a cell are captured. This motivates the use of dimensionality reduction methods tailored to count data to reduce the number of features and to smooth out random fluctuations. A common practice is reducing the data to a small number of latent dimensions (e.g., 50) prior to downstream computational analysis (Luecken and Theis 2019; Heumos et al. 2023).

Another motivation for single-cell representation learning is to capitalize on the large amount of available scRNA-seq data sets and identify expression patterns shared between them by mapping them into a single, common latent space. This is the idea underlying the development of foundation models for single-cell RNA-seq data (e.g., Theodoris et al. 2023; Cui et al. 2024), which are inspired by the success of foundation models in natural language processing (also called large language models). The hope is that by training on as many data sets as possible, the models will learn biologically relevant patterns, boosting their performance across various downstream tasks.

In this work, we refer to any approach transforming single-cell transcriptomic data from gene space into a latent space as *representation learning*. This is in line with Bengio et al. (2013), who defined representation learning as “learning representations of the data that make it easier to extract useful information when building classifiers or other predictors.” This definition applies to a broad range of scRNA-seq analysis methods, from linear factor models to transformer-based deep-learning models.

We compare different approaches to representation learning of scRNA-seq data, discuss benchmarks, and outline open challenges. While efforts to develop new representation learning methods have largely shifted to foundation models, a number of recent studies have shown that existing foundation models often do not outperform simpler approaches. We therefore believe it is important to clarify what capabilities should be expected, how they should be measured, and what baselines are appropriate for comparison. A central goal of this review is to identify conceptual relationships among representation learning methods, thereby facilitating future method development and comparison.

## REPRESENTATION LEARNING

### Definition of representation learning

**FIGURE 1.**
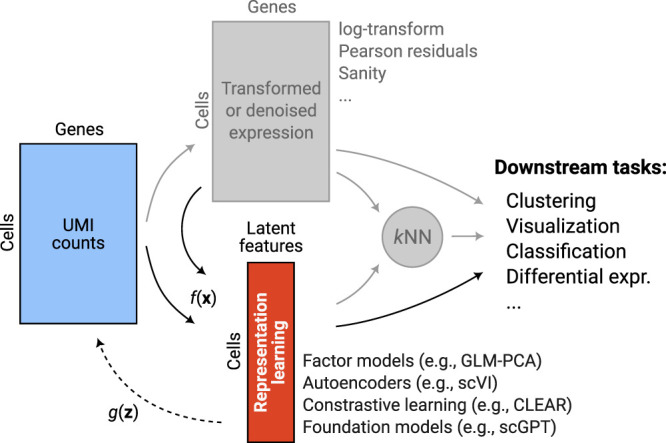
The role of representation learning in scRNA-seq data analysis. *k*NN stands for *k*-nearest neighbor graph, which is often used for further analysis.

The analysis of scRNA-seq data begins with a count matrix **X** of size *n* × *p*, where *n* is the number of cells, *p* is the number of genes, and a value *X*_*ij*_ denotes the integer number of RNA molecules coming from gene *j* detected in cell *i* (Fig. 1). In this paper, we will only talk about sequencing protocols based on unique molecular identifiers (UMIs) where each individual RNA molecule is counted at most once. Sometimes, cells may come from different *batches* (e.g., experiments using different sequencing technologies) with large technical variability between them; in this case, we denote as **b** a vector of length *n* storing the batch identity of each cell.

We define representation learning of scRNA-seq data as embedding each cell, for the purposes of downstream analysis, into some space ℝ^*d*^ with *d* dimensions. These *latent* dimensions do not anymore correspond to individual genes. The embedding is usually implemented by training the parameters of a function *f* that maps the gene expression profile of a cell *i* to a low-dimensional representation: **z**_*i*_ = *f*(**x**_*i*_), where **x**_*i*_ is the *i*th row of the matrix **X**. Typically, the dimensionality *d* of the latent space is chosen to be relatively low, for example, *d* = 50, and is almost always considerably smaller than the original dimensionality *p*. The resulting data representation is a matrix **Z** of size *n* × *d*. Some algorithms train a function that explicitly takes a cell's batch identity into account, that is, **z**_*i*_ = *f*(**x**_*i*_, *b*_*i*_).

We will discuss different approaches to train function *f* in the next section. Representation learning is usually understood to be unsupervised, meaning that the training is not informed by cell-type identities and the training algorithm only has access to **X** (and sometimes **b**).

Some representation learning methods work directly on the raw counts, whereas others expect the counts to be preprocessed. A standard preprocessing consists of two steps (Luecken and Theis 2019; Heumos et al. 2023): (1) accounting for the varying sequencing depths (the total number of counts can differ from cell to cell for technical reasons), by dividing the counts of each cell by a cell-specific size factor *s*_*i*_, and (2) applying a variance-stabilizing transformation such as the log-transform, to reduce the dependency of gene variance on gene mean (for count data, the variance typically increases with the mean). A related approach, combining both steps into a single one, is to convert the counts into Pearson residuals (Hafemeister and Satija 2019; Lause et al. 2021). We will denote a preprocessed count matrix using the same notation **X**.

We do not consider these transformations to constitute representation learning, as they preserve the original gene-level feature space. The same applies to data smoothing and denoising methods like MAGIC (Van Dijk et al. 2018) and Sanity (Breda et al. 2021), which are also outside of the scope of our review. Furthermore, we set aside methods that reduce the data to only two or three dimensions for visualization purposes. Such low-dimensional embeddings are not intended for downstream analysis (de Bodt et al. 2025) and are therefore not in scope of our review.

In the remainder of the paper, we discuss representation learning in the sense defined above: computational methods that transform gene counts into *d* latent dimensions, obtaining *d*-dimensional *embeddings* of each cell.

### The purpose of representation learning

The purpose of representation learning is to enable or facilitate downstream computational analysis (Bengio et al. 2013). The latent representation of a data set is hence only as good as it is practically useful.

Many representation learning methods have been claimed to be useful for a very wide variety of computational tasks. For example, Lopez et al. (2018) claim that their method, scVI, achieves high performance in “batch removal, normalization, dimensionality reduction, clustering, and differential expression.” Similarly, Cui et al. (2024) write that their method, scGPT, achieves state-of-the-art performance in “cell type annotation, genetic perturbation prediction, batch correction and multi-omic integration.”

Furthermore, at least for foundation models, the claim has been that this level of performance is achieved because, through training, the models acquire an implicit understanding of biology, similar to how large language models acquire an implicit understanding of language. The authors of Geneformer say that it “gained a fundamental understanding of network dynamics” (Theodoris et al. 2023), while the authors of scGPT write that it “effectively distills critical biological insights” (Cui et al. 2024).

The downstream tasks in single-cell data analysis are of a very different nature. Some are unsupervised tasks, such as clustering. Some are supervised, such as cell-type annotation, which can be done by training a supervised classifier on one data set and applying it to another. Differential expression analysis refers to statistical testing of differences in gene expression between prespecified cell populations. Perturbation prediction is another supervised task, where the goal is to predict the effect of a perturbation (e.g., a gene knockout, gene activation, or drug treatment) on the expression levels of other genes. A priori, it is unclear and may even seem unlikely that the same representation would perform well across such different tasks, and some methods address that by fine-tuning their representations for each downstream task separately. Moreover, for some of these tasks, it is even unclear whether they require any representation learning at all.

In the following sections, we will review and compare various approaches to representation learning and then discuss existing benchmarks of their performance in the above tasks.

## A REVIEW OF REPRESENTATION LEARNING METHODS FOR scRNA-SEQ DATA

**FIGURE 2.**
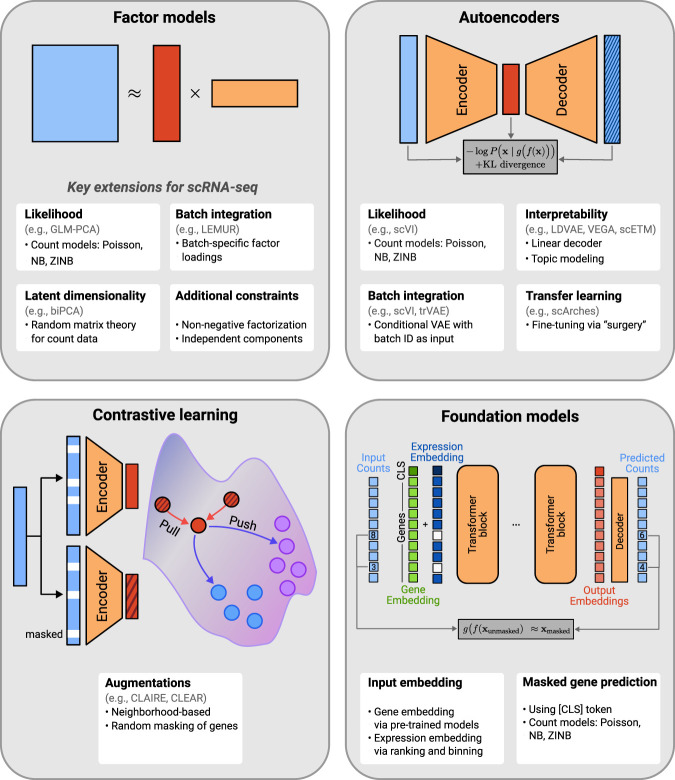
Conceptual illustrations of the four families of representation learning methods. (KL divergence) Kullback–Leibler divergence, (NB) negative binomial distribution, (ZINB) zero-inflated negative binomial distribution, (CLS) classification token.

We categorize the representation learning approaches into four groups: (1) factor models, (2) autoencoder-based models, (3) contrastive learning approaches, and (4) foundation models (Fig. 2). However, there are no strict boundaries between these groups, and methods from different groups can share important similarities. After reviewing the four groups of methods, we will propose a comparative taxonomy.

### Factor models

At the core of every factor model is the idea to decompose the matrix **X** into two lower-dimensional matrices, the latent representation matrix **Z** of size *n* × *d* and the loadings matrix **W** of size *d* × *p*, as follows:

X≈ZW.

Here, each cell's expression vector **x** (or its transformed version) is approximated by **W**^⊤^**z**, where **z** is the corresponding latent embedding vector.

The simplest example of a factor model is principal component analysis (PCA). It minimizes the reconstruction loss function,

$$L=\|\tilde{X}-ZW{\|}^{2},$$

where $\tilde{X}$ is the centered observation matrix, that is **X** after subtracting from each row the average gene expression vector **c**. The **ZW** term is the approximate reconstruction, and ${\left\|\cdot \right\|}^{2}$ denotes the sum of squared elements. Without loss of generality, the rows of **W** are constrained to be orthonormal. It turns out that optimal **W** has rows spanning the directions of maximal variance of the data. Furthermore, for this **W**, the optimal **Z** (the projection of the data into the space of principal components) is given by $\tilde{X}W^{⊤}$, and so PCA yields a linear function $z=f(x)=W\tilde{x}$ that can be used to compute the latent embedding of any new cell.

Reducing the dimensionality of the data is useful not only because it speeds up subsequent computations, but also because it can smooth out uncorrelated high-dimensional noise and thus avoid distortions of distances and neighborhoods (Li et al. 2025a). This perspective explains why *d* should be chosen large enough so that the model is sufficiently expressive but, at the same time, small enough so that undesired noise is smoothed out (Ahlmann-Eltze and Huber 2023). In practice, many scRNA-seq analysis libraries, such as Seurat, Scanpy, and the Bioconductor package scater, default to using 50 principal components. However, this is only a rule-of-thumb heuristic. A more theoretically grounded approach is given by biPCA (Stanley et al. 2025), which uses random matrix theory to estimate the number of principal components in a count matrix with Poisson or negative binomial observation noise. Depending on the data set, the authors obtain between 10 and 300 principal components exceeding the expected negative binomial noise.

In single-cell analysis, PCA is typically performed on normalized and log-transformed expression counts. PCA on Pearson residuals is closely related to correspondence analysis, a factor model for count data developed in the 1970s and recently applied to single-cell data (Hsu and Culhane 2023). A more advanced approach, avoiding this two-step procedure, is given by GLM-PCA, which is a matrix factorization method for raw integer counts borrowing ideas from generalized linear models (GLMs) (Townes et al. 2019). For one cell, the loss function of GLM-PCA is:

$$L=-\log P(x|s\cdot \exp(W^{⊤}z+c)),$$

where P(x∣μ) is the probability to observe the vector of expression counts **x** given predicted values *μ* under some count distribution, for example, Poisson or negative binomial. Note that the loss (negative log-likelihood) is calculated per cell and then summed up over all cells. The predicted expression values explicitly account for the average expression of each gene (**c**) and for the size factor *s* of each cell. The exponentiation ensures that the reconstructed values are nonnegative. Despite the elegant statistical formulation, GLM-PCA's adoption has been hindered by the challenging optimization (Lause et al. 2021; Ahlmann-Eltze and Huber 2023); however, there is ongoing work to improve the inference algorithms (Nicol and Miller 2025).

Factor models developed for scRNA-seq data in the 2010s, such as ZIFA (Pierson and Yau 2015) and ZINB-WaVE (Risso et al. 2018), often assumed a zero-inflated distribution for P(x∣μ). Later work showed that for UMI counts, a negative binomial model (i.e., an overdispersed Poisson model) fits the data well and that zero-inflation is not necessary (Svensson 2020). Sarkar and Stephens (2021) argue that the overdispersion observed in technical replicates is small, and the pure Poisson model can often be sufficient.

One limitation of all the above factor models is that the resulting low-dimensional cell representations are not adjusted for batch effects. This is most commonly resolved by applying a batch correction method like Harmony (Korsunsky et al. 2019) or Scanorama (Hie et al. 2019) to the PCA representation. However, this approach does not yield explicit functions to map back and forth between gene expression values and latent embedding. In contrast, the LEMUR model (Ahlmann-Eltze and Huber 2025) extends PCA by making the principal subspace **W** depend on the batch identity (*b*) of each cell, so that corresponding cell types are mapped to similar positions. The output of LEMUR is a batch-corrected embedding **Z** and a batch-dependent function *f*(**x**, *b*) that returns the latent representation conditional on the batch.

Several other flavors of factor models have been applied to scRNA-seq data. Nonnegative matrix factorization (NMF) restricts all elements of both **Z** and **W** to be nonnegative and can be applied directly to the nonnegative counts **X** without centering (Stein-O'Brien et al. 2019; Elyanow et al. 2020; Yerly et al. 2025). The nonnegativity constraints can make latent embeddings more interpretable: Gene expression values of each cell can be viewed as a weighted composition of multiple gene programs (Kotliar et al. 2019). Independent component analysis (ICA) aims to find a latent representation **Z** with statistically independent components, so that the decomposition becomes more interpretable (Saunders et al. 2018).

### Autoencoders

Autoencoders are a foundational architecture in modern deep learning, consisting of a nonlinear encoder function *f*(**x**) that maps the gene expression profile of a cell to a *d*-dimensional latent representation **z** (also called the *bottleneck*) and a nonlinear decoder function *g*(**z**) that reconstructs the expression profile from this latent representation (Hinton and Salakhutdinov 2006). When applied to scRNA-seq data, both functions are typically implemented as fully connected neural networks with one or several hidden layers. The model is trained by minimizing a reconstruction loss, essentially requiring that

x≈g(f(x)).

PCA can be seen as a linear autoencoder with both *f* and *g* constrained to be linear functions, and autoencoders can be understood as a nonlinear generalization of PCA.

Variational autoencoders (VAEs) assume a specific data distribution *p*(**z**) in the latent space and thus become generative models. In a VAE, one can sample a new latent vector **z** from *p*(**z**) and use it to obtain a new vector of gene expressions *g*(**z**). The VAE loss function combines the reconstruction loss of a regular autoencoder and a regularizing term (based on Kullback–Leibler divergence) that pushes the latent embeddings toward the prespecified prior distribution *p*(**z**), typically chosen to be standard Gaussian (Kingma and Welling 2013).

Following the general success of VAEs, multiple parallel works developed VAEs specifically for scRNA-seq data (Lopez et al. 2018; Eraslan et al. 2019; Grønbech et al. 2020). One of the most widely used methods is scVI (Lopez et al. 2018), which models the raw counts in a way similar to GLM-PCA:

L=−logP(x∣g(f(x)))+KLdivergence.

By default, scVI uses a 10-dimensional latent space and a zero-inflated negative binomial distribution for *P*, for which *g* predicts parameters including a mean expression. Zero-inflation is often considered unnecessary for modeling UMI counts (see above), yet the authors of scVI find that it works well in practice. scVI implementation allows to switch between Poisson, NB, and ZINB distributions.

A major challenge in scRNA-seq data analysis is the integration of data sets generated from different experimental batches, technologies, or laboratories, where technical variation can obscure biological signal. To address this issue, scVI conditions both the encoder and decoder on the batch ID:

L=−logP(x∣g(f(x,b),b))+KLdivergence.

Even though this loss function does not explicitly encourage batches to overlap in the latent space, in practice, it does typically lead to a shared latent representation across all batches, while batch differences are absorbed by parts of the model that have direct access to the batch ID *b*. In trVAE (Lotfollahi et al. 2020), the approach from scVI is extended by explicitly enforcing batch overlap in the loss function using a so-called maximum mean discrepancy regularization.

Grouping individual cells into distinct subpopulations is a common task in scRNA-seq data analysis. scMAE (Fang et al. 2024) tries to learn cell representations that enhance clustering performance by adopting a masked autoencoder strategy (He et al. 2022): It corrupts some fraction of gene expression values *X*_*ij*_ by shuffling them across cells and trains an autoencoder to predict which values were corrupted and to reconstruct the original expression matrix. Other approaches adapt VAEs to model cluster structure in single-cell data explicitly. scVAE incorporates clustering directly into the generative model by using a Gaussian mixture model prior in the latent space (Grønbech et al. 2020), enabling simultaneous dimensionality reduction and cluster assignment. scTree (Vandenhirtz et al. 2024) directly learns a hierarchical organization of cell types across different batches, providing an approach to model the hierarchical relationships between cell populations while accounting for technical variation.

VAEs are powerful because of their nonlinearity, but this comes at a cost: It is difficult to provide biological interpretations for individual latent dimensions. To address this limitation, several approaches introduced constraints on the decoder *g* that aim to enhance the interpretability. LDVAE uses a linear decoder while maintaining the nonlinear encoder of scVI (Svensson et al. 2020a). This asymmetric design enables direct interpretation of latent dimensions through the decoder's weight matrix, similar to loadings in PCA. The resulting model is similar to GLM-PCA but with an explicitly trained encoder (GLM-PCA itself does not train any encoder and directly learns embedding vectors). Building on this concept, VEGA (Seninge et al. 2021) incorporates prior biological knowledge by using gene-module-specific masks in the decoder, constraining the model to learn latent representations aligned with known biological processes. Similarly, scETM (Zhao et al. 2021; Chen et al. 2025) constrains the decoder using an approach inspired by topic modeling, effectively mapping latent dimensions to topics and then topics to genes, with topics informed by user-provided gene modules.

Several methods have used VAEs to leverage well-annotated reference data sets for cell-type classification and transfer learning. scGen (Lotfollahi et al. 2019) uses transfer learning for perturbation response prediction. scANVI (Xu et al. 2021) is a semisupervised variant of scVI that incorporates a categorical latent variable over the cell types, allowing assignment of unlabeled cells to cell types. However, conditional VAEs, such as scVI, explicitly use the batch identity *b* in their model architecture, making it impossible to apply pretrained models to new data sets (with unseen *b* values). To enable efficient adaptation of pretrained models to new data sets, scArches (Lotfollahi et al. 2022) freezes most parameters of a pretrained scVI model while only training the weights connected to a newly created batch variable. This allows new data sets to be projected into the pretrained latent space, where cell-type annotation can then be easily performed. scPoli (De Donno et al. 2023) conditions the encoder and decoder not directly on the batch identity, but on a learnable embedding vector of each batch, enabling population-level data integration and scArches-like transfer to new data sets.

While most of the above models typically operate on cells from a single species, scSpecies (Schächter et al. 2025) extends the approach of scArches to cross-species integration by allowing larger model changes during fine-tuning. SATURN (Rosen et al. 2024) takes a different approach by first using a protein language model to map protein-coding genes from different species into shared macrogenes, which are then used in an autoencoder framework. This enables SATURN to map cells from different organisms into a shared latent space for cross-species integration and cell-type label transfer.

### Contrastive learning

Contrastive learning has emerged in computer vision as a powerful self-supervised approach for learning semantically meaningful representations from unlabeled image data (Balestriero et al. 2023). The core principle involves creating multiple distorted views (also known as *data augmentations*) of each image through transformations such as cropping, flipping, and grayscaling and training the model to represent these views close together in the embedding space. Thus, the learned representations should be invariant to data transformations designed not to affect the semantic meaning of the image.

When applied to scRNA-seq data, contrastive learning methods work with cells instead of images. To illustrate how they work, we can use the example of SimCLR (Chen et al. 2020), applying its logic to transcriptomic data. SimCLR uses the InfoNCE loss function that encourages views of the same cell to get close together while simultaneously pushing apart views of different cells:

$$L=-\underset{(i,j)\in P}{\sum }\log\frac{\exp(z_{i}^{⊤}z_{j})}{\sum_{k\neq i}\exp(z_{i}^{⊤}z_{k})}.$$

Here (*i*, *j*) ∈ P are pairs of views of the same cell (also called *positive pairs*), **z**_*i*_ are normalized embedding vectors of each view, and the sum in the denominator goes over some subset of all views (sampled from the same training mini-batch). The numerator pulls **z**_*i*_ and **z**_*j*_ closer together, while the denominator pushes views of different cells apart, preventing the model from collapsing to a trivial solution with all inputs mapped to the identical representation. Other methods use various other mechanisms to avoid model collapse. For simplicity, we are calling all such methods *contrastive*, in the sense that they contrast between each other two views of the same cell (even though this terminology is not entirely standard).

An open question for contrastive learning of scRNA-seq data is which data augmentations to apply to gene counts. CLEAR (Han et al. 2022) uses augmentations that are supposed to simulate technical artifacts and noise characteristics of single-cell experiments. This includes randomly replacing some expression counts with zeros (to simulate possible zero-inflation), adding Gaussian noise, and randomly swapping some expression values between genes and between cells. scContrast (Li et al. 2025b) uses similar augmentations, but adds further supervised augmentations such as swapping expression values between cells of the same cell type.

CLAIRE (Yan et al. 2023) forms positive pairs by identifying mutual nearest neighbors between batches. It then creates an augmented view of each neighbor by mixing it with its within-batch nearest neighbors. This strategy aims to make batches overlap in the representation space. During training, CLAIRE identifies positive pairs that are difficult to pull together, assumes that they correspond to cells from different cell types, and removes these positive pairs from further training. Concerto (Yang et al. 2022b) generates positive pairs by using stochastic dropout layers in the network architecture, so that the network produces nonidentical embeddings for the same input cell (based on SimCSE, Gao et al. 2021).

The methods reviewed above are modeled after different contrastive methods from computer vision. scContrast follows VICReg (Bardes et al. 2022) in enforcing covariance constraints in the latent space to avoid model collapse. Concerto follows SimSiam (Chen and He 2021) in using a student and a teacher network to represent two views of each cell. CLEAR and CLAIRE both use the InfoNCE loss as in SimCLR and employ a training approach from MoCo (He et al. 2020).

CLEAR, scContrast, and CLAIRE use fully connected networks with two to three hidden layers as an encoder, while Concerto uses an attention-based teacher network. In computer vision, it is a well-established practice to remove the last layers of the network after contrastive training to improve the learned representation (Bordes et al. 2023), and scContrast and Concerto also use this idea.

### Foundation models

The term “foundation model” emerged during the rise of large language models (LLMs) and has since spread across many other domains, including single-cell biology. Foundation models are loosely defined as unsupervised models trained on a large amount of data that can be adapted for a variety of different tasks. Foundation models of scRNA-seq data are often trained on tens of millions of unlabeled cells, in some cases from multiple species (Yang et al. 2024; Adduri et al. 2025; Pearce et al. 2025). Such models promise to produce cell representations that can be used for many downstream tasks, sometimes without further fine-tuning (*zero-shot* usage).

While the idea of unsupervised pretraining does not imply a specific model architecture, most single-cell foundation models use the *transformer* architecture (Vaswani et al. 2017). As LLMs represent texts as sequences of word tokens, transformer models for single-cell data represent cells as sets of gene tokens (see Szałata et al. 2024 for a review of single-cell transformers). There are methods that use actual LLMs to encode genes and their expression through natural language (see Zhang et al. 2025 for a review). However, most methods represent the identity and expression of each gene as a numerical input embedding vector (typically 500- to 1000-dimensional). These embeddings of all gene tokens (plus the embeddings of optional tokens for cell metadata) are then further processed by multiple transformer blocks.

One transformer block consists of an attention layer, where token representations can affect each other, and a subsequent nonlinear transformation, where token representations are processed independently. This sequence repeats several times, refining the representation of each token. The final layer of the transformer outputs gene embeddings, which is a vector for each input gene in the context of all other genes in the cell. This is a by-product of embedding each gene separately and enables gene-level downstream tasks. In addition to gene tokens, many models use a CLS token (short for classification) that represents the input cell as a whole, enabling cell-level downstream tasks. If the CLS token is not used, the final gene embeddings for a cell can be averaged to obtain the cell representation.

One important design choice in a foundation model is how it encodes the identity and expression of each input gene (Haber et al. 2025). To encode gene identity, some methods, such as scGPT (Cui et al. 2024) and Geneformer (Theodoris et al. 2023), simply learn an embedding vector for each gene. Other methods, such as scBERT (Yang et al. 2022a), UCE (Rosen et al. 2023), and GeneCompass (Yang et al. 2024), directly use gene embeddings from other methods, like the protein language model ESM2 (Lin et al. 2023) or the co-expression model gene2vec (Du et al. 2019), thereby incorporating previous knowledge about each gene.

The embedding of gene identity is usually summed with the embedding of the corresponding gene expression *X*_*ij*_, with multiple strategies used to construct the expression embedding. Some methods, such as Geneformer and GeneCompass, order gene tokens in each cell by expression and encode a gene's rank via a positional encoding; Geneformer uses an additional normalization step to downweight housekeeping genes that are highly expressed across many cells. UCE does not use any explicit expression encoding, but samples genes with replacement according to their expression strength. Methods like scBERT, scGPT, and scMulan (Chen et al. 2024) bin expression values and embed the bin index. Other methods, such as scFoundation (Hao et al. 2024), use a fully connected network to transform the gene expression vector of a cell into a fixed number of token embeddings. As a result, tokens do not correspond to individual genes in this setup. Finally, TranscriptFormer (Pearce et al. 2025) uses expression-aware attention, giving higher weights to higher-expressed genes when computing attention interactions.

Attention computations scale quadratically with the number of used tokens, so in order to reduce the computational cost, many models have restricted themselves to a certain number of most expressed or most variable genes, often around 1000–2000. However, architectural innovations like FlashAttention (Dao et al. 2022) or Performer (Choromanski et al. 2021) that accelerate attention computations can allow much larger input sets, and some models, for example, scBERT, use them to process over 20,000 genes per cell.

Another important design choice is the training strategy. Single-cell foundation models typically either rely on masked token reconstruction, as in BERT (Devlin et al. 2019), or on next-token prediction, as in GPT (Brown et al. 2020). In both cases, the information about certain genes is masked from the input, and the network is trained to reconstruct their expression from the remaining, unmasked genes:

$$g(f(x_{\mathrm{unmasked}}))\approx x_{\mathrm{masked}},$$

using transformer encoder *f* and decoder *g* that reconstructs counts from the output representation.

The most popular training setup is the masked token reconstruction. Here, a fixed fraction of the input gene tokens (often 10%–30%) is masked, and the model computes all representations without access to the masked genes. From these representations, the model has to reconstruct the true expression of the masked genes. Depending on the model, this task can be cast into different loss functions. Many models (e.g., GeneCompass, scMulan, and scFoundation) use the mean squared error loss to predict gene expression. Models with rank-based encoding often predict the identity of the masked genes with the cross-entropy loss. UCE uses the binary cross-entropy loss to predict whether a masked gene was expressed. If the CLS token is used to produce the cell embedding **z**, then the models are sometimes trained to reconstruct all masked genes from the CLS embedding (e.g., in UCE).

Next-token prediction training is inspired by generative LLMs that are trained to predict the next word of an incomplete sentence. In practice, this is implemented by causal attention masking, which ensures that every token can only access tokens earlier in the input sequence. However, as genes in a cell do not have a natural order, this setup requires imposing some ordering, for example, by ranking genes by expression. For example, tGPT (Shen et al. 2023) and Cell2Sentence (Levine et al. 2024) are trained to predict the identity of each gene from all higher-expressed genes. Some methods, such as scMulan and TranscriptFormer, use next-token prediction without imposing any gene sequence and randomize the gene order across training batches. The result is essentially equivalent to masked token reconstruction, but with a variable fraction of masked genes ranging from 100% to almost 0%.

Several modifications of these pretraining setups have been proposed. In read-depth-aware pretraining, introduced in scFoundation and adapted by scPRINT (Kalfon et al. 2025), the input may consist of downsampled counts, and the task is to reconstruct the original counts. This is signaled to the model by a special token that encodes the desired total number of counts in the output. Some methods combine masked reconstruction with additional supervised loss terms for predicting cell type or other cell metadata (e.g., scMulan and scPRINT) or sequencing technology (e.g., TranscriptFormer). Others use Poisson (TranscriptFormer) or zero-inflated negative binomial (scPRINT) likelihoods to account for count data statistics.

Some authors claim that their models learn representations that can be directly applied to downstream tasks (e.g., UCE, scFoundation, and scMulan). However, such zero-shot representations struggle to consistently outperform simpler baselines like deep autoencoders (Kedzierska et al. 2025). Many foundation models (e.g., scBERT and scGPT) are supposed to be fine-tuned, either to new data sets or to specific downstream tasks, such as cell-type annotation or perturbation response prediction. In a typical fine-tuning setup, a task-specific readout network is appended to the pretrained foundation model for supervised training. Usually, to reduce overfitting to the labeled training data, only the later layers of the foundation model are fine-tuned (Baek et al. 2025). However, even after such fine-tuning, foundation models cannot always outperform simple baselines (Boiarsky et al. 2024; Ahlmann-Eltze et al. 2025) (see below).

Besides using gene and CLS tokens, some models reserve additional tokens for cell-level metadata. TranscriptFormer has a separate token encoding sequencing technology and a separate loss term to predict the technology during pretraining. In contrast, scGPT only uses its batch token during batch-aware fine-tuning, where the model uses adversarial training to ensure that cell embeddings across different batches overlap (Ganin et al. 2016). Many other foundation methods do not give batches any special treatment, but still claim that their vast pretraining implicitly takes care of many batch effects (Rosen et al. 2023; Theodoris et al. 2023) (see below for benchmarks).

Some recent foundation models have combined ideas from different groups of methods. For example, STATE (Adduri et al. 2025) uses a transformer architecture with an autoencoder-like reconstruction loss and a conditional batch treatment, similar to scVI. scConcept (Bahrami et al. 2025) is a transformer-based contrastive learning model that creates two views of the same cell by masking it down to two disjoint gene sets. Similarly, CellLM (Zhao et al. 2023) uses dropout over genes to create multiple views for a contrastive loss and combines that with masked gene reconstruction. Mix-Geneformer and scPRINT use a similar mix of masking-based and contrastive objectives.

### A comparative taxonomy

For convenience and in line with existing conventions, the presentation above is split into four sections (Fig. 2). However, substantial conceptual overlap exists across these groups, and in practice, their boundaries are often fluid. To address this hybridization and to effectively conceptualize the landscape of scRNA-seq representation learning approaches, we suggest a taxonomy defined by five independent axes (Table 1). Fundamentally, each method trains a function *f*(**x**) that maps data from the gene space into the latent space. By analyzing how different methods define and train *f*(**x**), the following axes capture the core design choices, allowing for systematic comparison.

**TABLE 1.** Some examples of representation learning methods reviewed in the text

Method	References	Loss type	Function *f*	Batch treatment	Pretraining	Counts
PCA	Pearson 1901	Reconstruction	Linear	None	None	Log-norm.
GLM-PCA	Townes et al. 2019	Reconstruction	Implicit	None	None	Raw
LEMUR	Ahlmann-Eltze and Huber 2025	Reconstruction	Linear	Conditional	None	Log-norm.
scVI	Lopez et al. 2018	Reconstruction	Fully connected	Conditional	None	Raw
scArches	Lotfollahi et al. 2022	Reconstruction	Fully connected	Conditional	Pretrained	Raw
scMAE	Fang et al. 2024	Imputation	Fully connected	None	None	Log-norm.
CLEAR	Han et al. 2022	Contrastive loss	Fully connected	None	None	Log-norm.
CLAIR	Yan et al. 2023	Contrastive loss	Fully connected	Overlap	None	Log-norm.
scConcept	Bahrami et al. 2025	Contrastive loss	Transformer	None	Pretrained	Ranked
Geneformer	Theodoris et al. 2023	Imputation	Transformer	None	Pretrained	Ranked
scGPT	Cui et al. 2024	Imputation	Transformer	None	Pretrained	Binned
STATE	Adduri et al. 2025	Reconstruction	Transformer	Conditional	Pretrained	Log-norm.

For an explanation of the columns, see “A Comparative Taxonomy.” Examples were chosen to include well-known methods and also to cover a broad range of modeling choices.

#### Training objective

The training must follow a specific training objective, typically formalized as minimizing a certain loss function. There are several types of possible loss functions (which can also be combined).

1. Reconstruction error. One approach is to minimize the reconstruction error, that is, to require that
  g(f(x))≈x,
  where *g* is some decoder function that maps the latent representation back to the gene space. Factor models and autoencoders typically use this training paradigm.
2. Denoising and imputation. Another approach is to distort **x** into **x**′, for example, by adding noise or by setting the expression of some genes to zero (i.e., masking) and then optimizing the reconstruction of the undistorted cell:
  $$g(f(x^{\prime }))\approx x.$$
  This approach is used by denoising and masked autoencoders, and also by most transformer-based foundation models, where masking a set of genes can be seen as a distortion (the model is trained to impute the expression values for masked genes).
3. Contrastive loss. A third option is to distort **x** twice, obtaining **x**′ and **x**′′ as two distorted views of the same cell, and then minimize the discrepancy between the two representations:
  $$f(x^{\prime })\approx f(x^{\prime \prime }),$$
  that is, train the function to be invariant to distortions.

#### Model architecture

Machine learning models implement the mapping *f*(**x**) using different parameterizations and architectural designs. In this context, four main architectural classes are commonly used.

1. Linear function. Here, the latent space is simply a subspace of the original gene space, and *f*(**x**) = **Wx** for some matrix **W**. An example is PCA. The default dimensionality of the representation is often set to *d* = 50.
2. Fully connected network. A nonlinear function can be implemented via a fully connected neural network, also known as a multilayer perceptron (MLP). This is the architecture typically used by autoencoders and many contrastive learning methods. scVI uses *d* = 10 latent dimensions by default, while many contrastive methods choose *d* = 128.
3. Transformer. A nonlinear function is implemented via a transformer network, with genes playing the role of tokens in language modeling, and each gene getting its own embedding. Most foundation models use this architecture. They typically use representations with dimensionality *d* ∈ [500, 1000].
4. Implicit function. In some methods, the latent embedding vectors **z**_*i*_ corresponding to each cell vector **x**_*i*_ are learned directly, without training any parametrized function *f*. As a result, new cells cannot be transformed into the latent space without additional optimization. An example is GLM-PCA.

#### Batch handling

When the models are trained on data coming from multiple batches (experiments, technologies, etc.), this can be handled in different ways:

1. Batch-conditional models. The mapping function (as well as the decoder *g*) can directly take batch ID as input: **z** = *f*(**x**, *b*). This is an approach used in conditional autoencoders, so we call it *batch-conditional*. Examples are scVI and LEMUR, as well as some foundation models like STATE.
2. Enforcing batch overlap. The training objective can directly encourage different batches to overlap in the latent space, for example, using discrepancy-based penalty or adversarial training. An example is PCA plus Harmony or the fine-tuning of scGPT for batch integration.
3. Ignoring batches. The simplest approach is to not give batches any special treatment, in which case batches may end up strongly separated in the embedding space.

#### Pretraining and fine-tuning

Another axis of variation is whether the model is supposed to be applied out-of-sample to data that were not part of the training data.

1. No pretraining. Some models are trained from scratch on the data of interest. Most factor models, as well as standard autoencoder and contrastive learning applications, fall into this category.
2. Pretraining. Here, the model is trained (pretrained) on a large amount of data and subsequently can be directly applied to new data (zero-shot usage). This is the intention behind many foundation models. For a specific downstream task, pretrained models are often supposed to be fine-tuned. This can be supervised fine-tuning aimed at a given downstream task (as is the case for some foundation models like Geneformer, GeneCompass, and scGPT), but it can also refer to unsupervised fine-tuning for additional batches. An example of the latter approach is scArches.

#### Count transformations

A more technical distinction is in how the model uses the raw integer counts.

1. Raw counts. From the statistical point of view, the most principled approach is to use raw counts directly and employ count-based likelihoods, for example, the Poisson likelihood. Examples: GLM-PCA and scVI.
2. Transformed counts. Alternatively, a model can use log-normalized counts (or some other normalization), as is commonly done when using PCA.
3. Binned, ranked counts. Many transformer-based models make a more drastic step of binning counts into large bins (e.g., zero to 10 counts) or ranking all genes by expression and only predicting the rank.

Combining these modeling choices yields numerous plausible architectures, many of which appear in popular methods (Table 1), while others remain unexplored.

## BENCHMARKS AND PERFORMANCE COMPARISONS

Previous sections focused on conceptual differences between representation learning methods. However, what matters in practice is which methods work well for which specific tasks. When new methods are introduced, the authors usually provide some performance comparisons with previous approaches, but to be most trustworthy, a benchmark should be conducted independently.

The main challenge in benchmarking unsupervised representation learning for single-cell data is that there is no single performance indicator that can be assessed on held-out data. Instead, the quality of a representation can be measured in multiple ways and can depend on the intended *downstream task*. Many such tasks can be supervised (e.g., a classification task), which makes the comparisons easier but requires ground-truth labels. Such labels can be derived from another experimental assay, from expert annotations, or, when working with simulated data, can be known directly. Here, we survey some of the existing benchmarks, grouping them by the task they consider.

### Representation of biological structures

This task considers a single data set with no batch effects. What representation learning method captures biologically meaningful structures the best? One way to assess this is to cluster latent embeddings and compare clustering results with known cell-type labels. Germain et al. (2020) derived labels from manual annotation and from simulation and compared various factor methods and scVI, concluding that PCA on Pearson residuals worked the best. To move beyond discrete groups, one can use a simulation to test how often known nearest neighbors of a cell remain nearest neighbors in the embedding space. This approach was first suggested by Breda et al. (2021), who developed Sanity, a Bayesian denoising method without representation learning, and found that it outperformed scVI in this task. Ahlmann-Eltze and Huber (2023) used a similar approach and showed that PCA on log-transformed counts performed as well as, or better than, more complex approaches, and demonstrated the importance of using a reasonable number of principal components.

### Batch integration

This task evaluates to what extent the methods remove known batch effects while preserving the biological structure. Here, batch labels serve as the ground-truth to test if cells from different batches are intermixed. This performance metric has to be balanced with another metric that captures preservation of biologically relevant structures, usually cell types (often relying on the cell-type annotations from the original publication). Luecken et al. (2022) found that for integrating data sets with complex conditions, Scanorama and scVI performed the best among unsupervised approaches (that do not use cell-type labels during training). They also included supervised methods (e.g., scANVI) in the comparison, but the cell-type supervision arguably gives those an unfair advantage. Maan et al. (2024) found that scVI and Harmony performed well, even under strong cell-type imbalance.

Ovcharenko et al. (2025) compared contrastive learning methods, VAE methods, and foundation models and found that scVI, CLAIRE, and batch-aware fine-tuned scGPT performed the best. In contrast, zero-shot scGPT performed very poorly at batch integration. Kedzierska et al. (2025) reached the same conclusion for zero-shot scGPT and Geneformer. Ovcharenko et al. (2025) also did a careful ablation of augmentation strategies for contrastive learning and demonstrated that random masking was the most important augmentation for batch integration. Liu et al. (2024) analyzed the performance of several fine-tuned foundation models and found that Harmony typically performed better.

### Cross-species integration

A related task is cross-species data integration, where between-species differences play the role of a batch effect. Zhong et al. (2025) found that, among cell-type-unaware methods, SATURN and scVI perform well for this purpose. SATURN achieved the strongest overall performance, while scVI was especially effective in the presence of strong batch effects. Song et al. (2023) showed that Harmony and Seurat's explicit batch correction method performed the best, followed by scVI. The supervised method scANVI achieved the best score.

### Cell-type prediction

This is a standard classification task that refers to assigning cell-type labels to the test data based on some labeled training data set, using, for example, a linear or a *k*NN-based classifier. The test data can be substantially different from the training data, for example, from a different donor or species. Richter et al. (2025) compared contrastive and masked autoencoder methods (implemented by the authors) to a supervised classifier. Representations were pretrained on a large data set of over 20 million cells with self-supervised learning objectives. For the in-distribution test data, the authors found that after supervised fine-tuning, the self-supervised pretraining did not improve the predictions compared to supervised training. Without fine-tuning, masked autoencoders performed better than contrastive learning methods, but much worse than after fine-tuning. The authors also showed that on smaller (as well as out-of-distribution) test data sets, pretraining followed by fine-tuning outperformed a supervised classifier trained from scratch.

Boiarsky et al. (2024) compared scGPT and scBERT, fine-tuned for classification, with regularized logistic regression trained on the same data. In this setup, there was no distribution shift between the test set and the training set (also used for fine-tuning). The authors found that logistic regression performed as well as foundation models, even when the training set was small. Liu et al. (2024) used a similar setup, but found that fine-tuned foundation models, in particular scGPT, outperformed a simple support vector machine classifier. On the other hand, Fu et al. (2024) did a similar comparison using a data set with ground-truth labels derived from cell sorting and found that SVM performed the best, followed by fine-tuned scBERT.

### Perturbation prediction

In perturbation experiments, the expression of one or several genes is changed (e.g., through knockouts or knock-ups), and the effect on the expression of all other genes is measured. Foundation models have been used for predicting perturbation effects by setting a gene's expression to zero in silico and measuring the predicted expression of other genes. However, a large number of benchmarks showed that foundation models do not yet outperform simple linear baselines for predicting the effect of unseen single or double perturbations (Bendidi et al. 2024; Kernfeld et al. 2024; Wu et al. 2024; Ahlmann-Eltze et al. 2025; Csendes et al. 2025; Wenteler et al. 2025). More recently, several benchmarks aimed to provide a more nuanced assessment by investigating metrics where foundation models do perform better (Miller et al. 2025; Viñas Torné et al. 2025; Wei et al. 2025), and we feel the jury is still out on this matter. For benchmarks to be relevant in practice, it is important that the metrics are chosen based on the intended application.

## CHALLENGES AND OPEN ISSUES

The preceding sections highlighted several unresolved issues in scRNA-seq representation learning. Here, we outline the key challenges and open questions that we consider the most critical for the field.

### 1. When is representation learning needed?

Different tasks, such as cell typing and trajectory inference, may require distinct representations. However, some tasks may not require representation learning at all. For example, identifying differentially expressed genes is a question inherently formulated in the gene space and, arguably, should not be analyzed in the latent space. Likewise, cell typing can be carried out without representation learning, even under strong batch effects (e.g., Scala et al. 2021). In addition, rather than attempting batch-effect correction, it may be more appropriate in some cases to analyze each batch separately.

### 2. Do single-cell transcriptomic data have low intrinsic dimensionality?

There are two arguments in favor of reducing the dimensionality of scRNA-seq data. First, although the true intrinsic dimensionality is unknown, it is likely far smaller than the total number of genes. Second, very high-dimensional noise (such as noise in the gene space) distorts distances because of the curse of dimensionality (Li et al. 2025a), which dimensionality reduction can mitigate. On the other hand, any dimensionality reduction and representation learning comes at the cost of replacing physically meaningful quantities (gene expression activities) with noninterpretable, *latent* features.

Notably, the methods reviewed above tend to use very different dimensionalities. scVI-like autoencoders usually set *d* = 10 by default. Factor models like PCA are often used with *d* = 50. Many contrastive methods choose *d* = 128, and foundation models often have *d* = 768. These differences may need to be accounted for to ensure fair method comparison.

### 3. What is the necessary model complexity?

How complex does the mapping *f* need to be for scRNA-seq data? Scaling laws in natural language modeling suggest that, when trained on enough data, larger models outperform smaller ones (Hoffmann et al. 2022), and the largest existing LLMs contain over 1 *trillion* parameters. But whether the same is true for scRNA-seq is unclear and depends on the inherent complexity, redundancy, and availability of scRNA-seq data. Recent foundation models saw a huge increase in complexity compared to prior models—for example, scGPT has 50 million parameters and 12 transformer layers, and UCE has 650 million parameters and over 30 transformer layers, whereas scVI models typically have only 1–2 million parameters in one to two hidden layers—but whether this model complexity is necessary for good performance is largely unclear. The field should investigate scaling laws on well-defined benchmarks.

### 4. How to incorporate statistically accurate count modeling?

Some representation learning approaches, such as GLM-PCA, biPCA, and scVI, emphasize modeling raw UMI counts using appropriate count distributions. In contrast, many foundation models, such as scGPT, rely on binned counts and squared error losses. Only recently have foundation models such as scPRINT begun to incorporate more statistically principled likelihoods. A key open question is when and to what extent such likelihood-based objectives improve performance in advanced architectures, including large foundation models, relative to simpler strategies.

### 5. How should we benchmark scRNA-seq representation learning?

Benchmark efforts in the field of scRNA-seq representation learning face many challenges. First, some of the existing benchmark metrics are problematic (Rautenstrauch and Ohler 2025) and can even be gamed (Wang et al. 2025). Second, systematic comparisons across method families, for example, foundation models versus autoencoders, remain underexplored. When performance differs, it is often unclear whether this is due to training-data volume, model size, architectural choices, or other factors. Third, it is very important to use strong and appropriate baselines, as recent work on perturbation prediction has demonstrated. Finally, various benchmarks compare different methods with little overlap, and static benchmarks can quickly become outdated as the number of new methods grows. Therefore, the community would benefit from continuous benchmarks such as the Open Problems initiative (Luecken et al. 2025) and the Omnibenchmark project (Mallona et al. 2024).

### 6. How to make ablation studies more popular?

While benchmark studies focus on black-box comparisons of published and packaged tools, ablation studies use an open-box approach, taking models apart to see which components really matter for performance. Ablation studies are very important for progress in the field, and more attention should be devoted to them. Some recent examples include comprehensive ablation studies of the modeling choices in scRNA-seq foundation models (De Waele et al. 2025; Haber et al. 2025). When pretrained foundation models are used as components of larger multimodal models and subsequently fine-tuned, it is also important to perform ablation studies to investigate the roles of pretraining and model architecture.

### 7. How to avoid data bias when training foundation models?

The generalization ability of foundation models hinges on the amount and diversity of the pretraining data, which often pools tens of millions of cells. The challenge lies in ensuring that this vast pool is truly representative across tissues, disease states, and experimental conditions. Bias in pretraining data can limit the model's performance on novel, underrepresented samples, making the learned representation less universal than claimed.

## CONCLUSION

In this paper, we set out to clarify the landscape of representation learning methods for scRNA-seq data by reviewing four major paradigms: factor models, autoencoders, contrastive approaches, and transformer-based foundation models. By articulating the core modeling choices shared across these methods and surveying existing benchmarks, we aimed to provide a clearer understanding of the strengths and limitations of current approaches. Rather than offering definitive answers, we identified and discussed open questions, such as whether data augmentation or masked prediction is more effective, whether large-scale pretraining is necessary for specific downstream tasks, and whether transformer architectures offer meaningful advantages over fully connected networks in this domain.

We believe that large foundation models trained on the vast amount of collected single-cell data have promise and potential. At the same time, current models often claim to learn “universal representations” (Rosen et al. 2023), extract “fundamental knowledge” (Theodoris et al. 2023) and “generalizable feature[s]” (Cui et al. 2024), but often struggle to outperform simpler methods in the benchmarks we reviewed. In our view, declaring victory too early may hold the field back. Instead, we call for comprehensive ablation studies and evaluations with strong baselines, which can help identify ideas truly foundational to learning representations of single-cell data.
